# Principles Involved in Filling Teeth with Gold Foil

**Published:** 1877-08

**Authors:** S. B. Palmer


### ARTICLE III.

### *Principles Involved in Filling Teeth with Gold Foil.*

BY S. B. PALMER, M. D. S.

Read before the fifth District Dental Society of the State of New York, April 24th, 1877.

The late History of Dentistry shows, that filling teeth with gold has furnished a fruitful topic for discussion from the earliest use of the metal for that purpose, down to the present time.

The earnestness displayed in discussion and the various preparations of gold recommended, show most conclusively that we are from the millennium of a uniform practice. The filling of a tooth involves known principles in nature, which, in all cases, determines the result of the operation. Failing to recognize such a law, time, reputation and teeth are sacrificed; and ever will be, until these principles are complied with. This lack of knowledge is very natural. Dentistry, but a few years since, was only a mechanical employment; its advance to a profession is mainly the result of observation, and experiment. By this means, rather than from scientific investigation, is dentistry indebted for the facts and knowledge that concedes to it the honor of a profession.

Although dentistry claims to be a profession, in the one operation of filling teeth, the parental type or mechanical inheritance is predominant; even some of our best operators say that filling teeth is a "mechanical operation, no chemistry about it, that failures are due to miserable mechanics." If it had been the good fortune of the writer to find the perfect mechanic, and we judge them by their work, this article would not have been written. It is not my purpose to give any one method of operating or preparation of material to be used in the preservation of teeth. There are two considerations in filling a cavity in a tooth. First mechanical, second chemical.

Manipulating the gold, and finishing the plug, is mechanical. We have been told how to do this time and again, and by all conceivable methods, and still every operator has his black list, which he does not care to exhibit. And why? Because the effort was against natural laws, and could not succeed. When the profession come to understand the conflict between the chemical forces, set in action by the presence of gold in contact with dentos, and efficiency of the plug to mechanically check those forces, will there be less failures, because there will be less gold fillings of that class inserted. Having said this much for mechanics, let us consider the true causes of anxiety and disappointment, that dentists so often meet with in practice. In a word they undertake impossibilities.

Without going over the premises occupied by other papers, I will only mention facts, as acknowledged and understood by modern scientists, relative to matter, or in a strict sense, matter with which we have to do in filling teeth with gold. It is true that gold, not in absolute contact with a tooth, worn in the mouth as filling or clasp, sets in action one or more of the correlated forces of nature, to the detriment of the tooth, in a degree, according to the resistance of the tooth. Heat, chemical affinity, and electricity are three of the forces most active in this connection. Moisture is the medium or agent through which this destructive action does its work. Perfect contact of gold and dentos excludes moisture and hence no appreciable decomposition takes place. This fact calls for thoroughnes in removing all softened bone from the cavity, and close adaptation of the gold to the dentos.

That power of force called capillary action which raises the giant forest tree above the surface of the earth, in opposition to gravitation, is a potent agent in causing the destruction of teeth filled with gold. Take a tooth that has been extracted and become dry, place it in water so that a small portion will be immersed, the whole body will soon be dampened by capillary action. Teeth in the mouth are always more or less moist according to their density, the fine scratches left by the burs, and excavators in preparing cavities, are not, and indeed cannot be perfectly filled with ordinary foil, and therefore become filled with the fluids of the mouth, the very thing neccessary to call into action, and conduct the electric current caused by the presence of gold. This may be considered the general cause of enlargement of cavities, and loosening of gold plugs. Perfect fillings will preserve good teeth; nature protests against the use of gold in teeth sufficiently porous to become conductors of the electric current, her laws are imperative, only in their keeping can we expect reward. Much time is wasted in experimenting, and discussing the various methods, and preparations recommended; that there is a vast difference in the skill of operators I am ready to grant. That preparation of gold is the best for the dentist which will enable him to most perfectly fill a cavity; when all is done, gold is gold, and all the thoroughness, and skill at our command cannot change its natural influence upon tooth substance in the mouth. While I deplore the use of the file for separating teeth and admire the beautiful results attained by wedging and contour filling, I am convinced that the advantages gained by the latter course lie in admiration, and not preservation, for the same principal governs the action when a contour filling touches an approximate tooth, as when a clasp is in contact or a filling loose in a cavity, but for the enamel upon the approximate tooth to check the action contour fillings would soon fall into disuse.

The object in presenting this subject, is not to bring general charges against the use of a material which must still be employed for want of better, but to present the principles which will ever determine the result for good or evil. These principles are ours for the finding out, and are as unchangeable as nature. To warrent success, cavities must be prepared with brains as well as by burs and excavators; gold must be packed against the walls of the cavity with good judgement as well as by the mallet. I am convinced that the bruising of the dentos by over-packing hard gold upon the surface is one of the causes of many unaccountable failures witnessed. The success of many of the early dentists in the use of gold, lay not in their superior skill, but rather in conformity to laws granting success. The average operator of to-day would be as successful with permanent separations, and non-cohesive gold. In our desire to restore the wasted portions of teeth cohesive gold is used, with which it is at times impossible to do perfect work; and again, instead of the permanent separation, there is often contact with an opposite tooth or filling, failure is only a question of time. It is not my intention to advocate the old practice; I would that all might know the principle by which to be governed, or which will surely govern any operation; then with judgement choose that method which under all circumstances, would give the best results. It has been but a few years since the relation of matter and force has attracted attention in filling teeth; up to this period we had all the facts or effects which had come through patient observation and experiment during the existence of the profession. Given those effects, by the aid of science we go back in search of causes, having found them, we call them principles or laws, so far as they relate to our specialty. They are not numerous or difficult of comprehension, and ere long the dental student will be taught the same as the rudimental principles in the practice of dentistry. Let those doubt who may, science never put her hand to the plow to look back.—*Dental Register.*

